# The operation of a Research and Development (R&D) program and its significance for practice change in community pharmacy

**DOI:** 10.1371/journal.pone.0184954

**Published:** 2017-09-18

**Authors:** Andi Hermansyah, Erica Sainsbury, Ines Krass

**Affiliations:** 1 Faculty of Pharmacy, the University of Sydney, New South Wales, Sydney, Australia; 2 Faculty of Pharmacy, Airlangga University, Surabaya, Indonesia; University of Mississippi, UNITED STATES OF AMERICA

## Abstract

**Background:**

Community pharmacy practice in Australia is changing and Research and Development (R&D) in community pharmacy plays an important role in contributing to the changes. A range of Cognitive Pharmacy Services (CPS) were developed from R&D programs, yet their implementation has been minimal indicating slow practice change within community pharmacy. Given the vital role of R&D, little is known about the operation and the extent to which it has been effective in supporting practice change in community pharmacy.

**Methods:**

In depth, semi-structured interviews were conducted with 27 key stakeholders in the pharmacy and healthcare system in Australia. All interviews were audio-recorded, transcribed ad verbatim and analysed using an inductive approach.

**Results:**

Participants perceived that the R&D program has played an important role in the advent of CPS. Furthermore, they considered that evidence generated by the R&D projects is a critical influence on policy formulation, funding and implementation of CPS into practice. However, policy decisions and subsequent implementation are also influenced by other factors associated with context and facilitation which in turn foster or inhibit effective Knowledge Translation (KT) in the community pharmacy sector.

**Conclusion:**

While R&D programs have been viewed as essential for supporting changes in community pharmacy practice through development and funding of CPS, the overall impact has been small, as contemporary practice continues to be predominantly a dispensing model. Given the complexity and dynamic nature of the community pharmacy system, stakeholders must take into account the inter-relationship between context, evidence and facilitation for successful KT in community pharmacy practice.

## Introduction

Community pharmacy practice is under pressure to change. Over recent years, the revenue generated from dispensing prescriptions has become constrained, profit margins are falling and the sales of non-pharmaceutical products are diminishing [1–4]. There is a clear imperative for community pharmacies to change their business model beyond dispensing and sales of pharmaceuticals [3,5,6]. Internationally, there is a growing body of evidence which shows that community pharmacy has increasingly turned to providing expanded health-related services as a revenue stream to offset the losses from traditional dispensing practice [7–9].

With regards to the practice change paradigm, Research and Development (R&D) in community pharmacy has been increasingly acknowledged as a driver for the development of new Cognitive Pharmacy Services (CPS). In Australia, R&D in community pharmacy has been funded through the consecutive Community Pharmacy Agreements (the CPAs) which are five-year agreements between the Australian government and the Pharmacy Guild of Australia. Commencing in 1990, the agreements have provided funding of over $45 billion to support a viable network of community pharmacies throughout Australia including funding support for the R&D program (Table 1) [10].

**Table 1:** Proportion of funding under the consecutive CPAs.

	1^st^ CPA (1990–1995)	2^nd^ CPA (1995–2000)	3^rd^ CPA (2000–2005)	4^th^ CPA (2005–2010)	5^th^ CPA (2010–2015)	6^th^ CPA (2015–2020)
Total funding^a^, (% increase from previous CPA)	$3.286 billion	$5.497 billion (↑67%)	$8.804 billion (↑60%)	$12.158 billion (↑38%)	$15.610 billion (↑28%)	$18.886 billion (↑21%)
Funding for Pharmacy Remuneration^b^, (% within the CPA)	**Not Available**	**Not Available**	$5.6 billion (63%)	$11.1 billion (91%)	$13.8 billion (89%)	$14.8 billion (78%)
Funding for CPS, (% within the CPA)			$114 million (1.29%)	$241 million (1.98%)	$427 million (2.77%)	$368 million^c^ (1.94%)
Funding for R&D, (% within the CPA)		$5 million (0.1%)	$15 million (0.17%)	$19 million (0.16%)	$11 million (0.06%)	$50 million^d^ (0.26%)

^a^Actual expenditure under each agreement was not publicly reported. These numbers were obtained from the Audit Report on the Administration of the 5^th^ CPA;

^b^Funding that was related only to payment for supplying the medicines e.g. dispensing fee, pharmacy mark-up, premium fee dispensing incentives, extemporaneous preparation etc.;

^c^Approximately $613 million was invested in the 6^th^ CPA under funding for Community Pharmacy Programs. However, only half of this funding was allocated for provision of professional pharmacy services as indicated in Appendix B of the agreement. Additional funding up to $600 million will be provided based on the recommendations of the Health Technology Assessment Body after evaluating the outcome of the Pharmacy Trials Program.

^**d**^ Funding for the R&D program was ceased in the 6^th^ CPA and shifted to fund the Pharmacy Trial Program.

**Fig 1:**
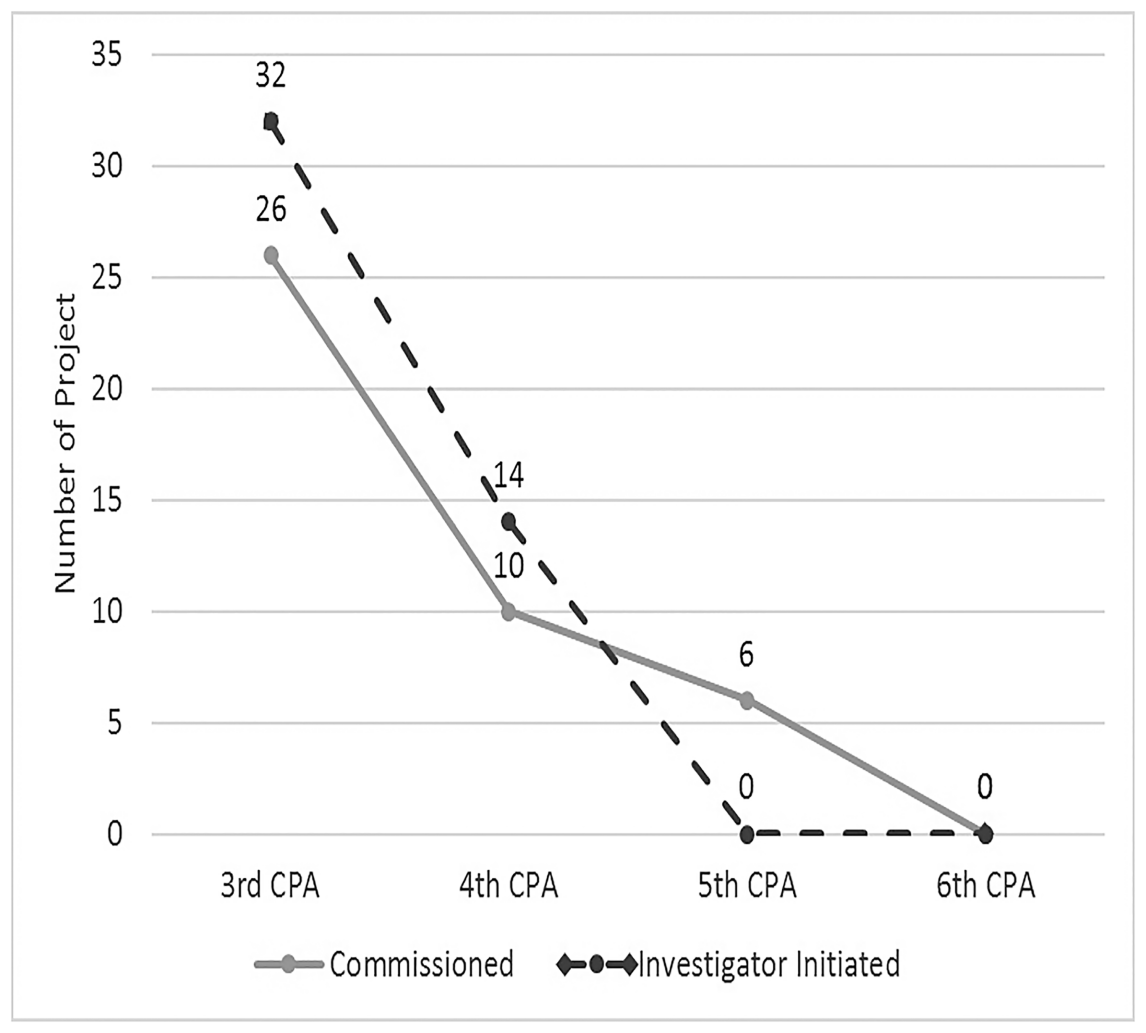
Distribution of R&D projects under the CPAs.

The R&D program funded two types of projects (Fig 1): Investigator Initiated Grants (IIGs) and Commissioned projects. In the IIG, researchers designed projects aligned with their own research or interests within each CPA. On the other hand, Commissioned projects were announced for public tender with the research program already pre-determined by an Expert Advisory group encompassing key stakeholders in pharmacy [11,12].

Despite the importance of the R&D program, there has been concern expressed about the way the program has been funded, operated and its effectiveness in supporting practice change in community pharmacy. For instance, only a very small proportion of the total CPA funding was invested in the R&D program with the majority having been allocated to fund the supply of medicines including the allocation for dispensing fees of Pharmaceutical Benefits Scheme (PBS) medicines (Table 1). In fact, the amount of funding for R&D fell steadily from the 3^rd^ CPA onwards, and was ceased in the current CPA (the 6^th^). However, there was funding provided to trial new and expanded community pharmacy services under the Pharmacy Trial Programs. Furthermore, there was a trend within the most recent agreements towards funding Commissioned projects which limited opportunities for independent innovative research in the community pharmacy sector (Fig 1).

### From research to practice: The case of DMAS, PAMS and HMR

The implementation of CPS involves a long and complex process from the design and development of the research, to evaluation of the impact on clinical, humanistic and economic outcomes of a variety of CPS, dissemination of the research findings to stakeholders, to the adoption and implementation which leads to sustainable delivery of CPS in community pharmacy. In the R & D phase of previous CPAs there was scant attention paid to development of a strategy to achieve knowledge translation using principles of implementation science.

Within this section, we describe three case studies of pharmacy services funded under R&D program of the consecutive CPAs. They are Diabetes Medication Assistance Service (DMAS), Pharmacy Asthma Medication Service (PAMS) and Home Medicines Review (HMR). These services were trialed using rigorous research designs i.e. randomized controlled trials, thereby providing a body of sound evidence of benefit in community pharmacy. While these benefits supported the possibility for larger scale implementation, the policy decision was not to proceed with the implementation of these services in community pharmacy reflecting the long process and complexity for developing R&D in community pharmacy.

The Diabetes Medication Assistance Service (DMAS) and Pharmacy Asthma Medication Service (PAMS) were two research projects examining the clinical and cost effectiveness of chronic disease management support services provided by community pharmacists for patients with type 2 diabetes and asthma respectively [13,14]. These services were initially funded as small trials, and subsequently tested in randomized controlled trials in the 3^rd^ CPA. Under the 4^th^ CPA, the funding for DMAS and PAMS was continued for national pilot programs. Despite the success of the pilot programs, funding for neither of these CPS was continued in the 5^th^ CPA.

When it was funded for small trials as part of the Pharmacy Diabetes Care Program under the 3^rd^ CPA, DMAS was found to be operationally and clinically effective [15,16]. Under the 4^th^ CPA, the service was then rolled out for a two-stage pilot program, first to 90 pharmacies and then 800 pharmacies across Australia. In the first stage, the benefit of DMAS was demonstrated in terms of improved clinical outcomes and acceptance by patients, pharmacists and GPs [13,17,18]. However, the uptake of the service in stage 2 was limited. The evaluation of stage 2 DMAS identified operational problems such as poor interaction with other health professionals including GPs, difficulties in recruiting patients which were predominantly related to lack of interest from patients, limited time and capacity of pharmacists to provide the services and mixed patient health benefits with only marginal improvement in clinical outcomes and lifestyle factors [19]. As a result, continuation of DMAS in its original form was not considered to be necessary or economical [19].

In the 5^th^ CPA, DMAS was modified into Diabetes Medscheck, a one off meeting with a pharmacist to review the medication and management plan for a patient with type 2 diabetes [20]. The modification removed the most valuable part of DMAS which was patient support and monthly monitoring to facilitate better self-management for patients with established diabetes [21]. The Evaluation report on Medscheck and Diabetes Medscheck demonstrated mixed perceived benefits. Although there was an increase in consumers’ knowledge about their medication regimens, the programs may not have reached their targeted patients such as high-risk patients, patients with chronic diseases, and patients taking multiple medications. In addition, in contrast to DMAS and PAMS there was no evidence demonstrating clinical and cost-effectiveness of these services to justify their funding [22].

Research for PAMS originated from the Pharmacy Asthma Care Program (PACP), also funded under the 3^rd^ CPA. The research demonstrated significant improvement in asthma control and quality of life of patients with asthma and the service was found to be cost effective [23]. The project was rolled out as PAMS for a pilot program under the 4^th^ CPA, and involved several consultations with the pharmacist in the pharmacy over a period of 6 months. The service included assessments of asthma severity, inhaler technique, medication adherence, the use of spirometry, patient counselling on asthma triggers, goal setting and referral to GPs as appropriate [24,25]. The pilot was initially designed in two stages. The evaluation of stage 1 which involved 100 pharmacies showed that clinical outcomes and substantial economic efficiencies would be achieved if PAMS were implemented in a broader community pharmacy setting [26]. However, after stage 1 was concluded, a decision was made by the Department of Health, in consultation with the Pharmacy Guild, not to proceed with stage 2 [26]. There was no explanation publicly available concerning the reason for not continuing the PAMS project.

Even the implementation of reasonably well-established CPS such as Home Medicines Review (HMRs) has not been without problems. HMRs have been part of CPS in Australian community pharmacy since 2001 and have proven to be sustainable to date. HMRs are provided jointly by doctors and accredited pharmacists specifically for patients who may benefit from a medication management plan. Research on HMRs has shown that it is an evidence based and cost effective service that prevents and resolves medication related problems [27–29]. In addition, there is evidence that HMRs are cost saving to the healthcare system [30].

Funding for HMRs was initially allocated under the 3^rd^ CPA, however the increasing uptake of the services since 2002 has resulted in growth in demand significantly outstripping the funding allocation [31,32]. This was particularly evident during the 5^th^ CPA, when funding for HMRs was exhausted before the conclusion of the Agreement. The decision not to provide additional funding for the service reduced the effectiveness of the service, affected particularly vulnerable patients and threatened the sustainability of medication management programs in the future [22,33].

Stakeholders’ thoughts and opinions about the operation of the R&D program and its significance for practice change have not yet been explored in the literature. Therefore, this study aimed to analyse the operation of the R&D program funded under the Community Pharmacy Agreements and its impact on knowledge translation in Australian community pharmacy practice.

## Materials and methods

In-depth, semi structured interviews with a wide range of key stakeholders within and beyond community pharmacy were employed to address the aim of this study. The participants represented multiple actors in pharmacy and the healthcare system including practicing pharmacists, professional peak pharmacy and medical organizations, GPs, consumer organizations, private insurance companies and the government. The participants also represented both genders, different States within the eastern half of Australia (Queensland, Australian Capital Territory, New South Wales, Victoria and South Australia), various pharmacy backgrounds (banner pharmacy group, discount chemist and sole proprietor) and metropolitan to rural areas.

The participants were firstly selected using purposive sampling and the snowball sampling method was used to expand the initial sample; at the end of each interview, participants were asked to nominate other potential candidates for the study. Face to face interview was the interview method of choice, but a number of participants were interviewed by telephone and Skype video. Written consent was obtained from participants prior to the interviews. This study was approved by the Human Research Ethics Committee of the University of Sydney.

The interview was based on a series of key questions that were developed from the literatures on R&D and implementation science, the contemporary situation of pharmacy practice in Australia and discussions among investigators. Several literatures highlighted the complexity and variation in the implementation process including the presence of both opportunities and challenges from which the investigators developed the interview guide [34–37]. The guided questions were piloted with three different key stakeholders and revised based on their feedback. There were no changes concerning the content of the interview questions. Improvement was made in relation to wording, order of the questions and how to probe respondents’ comments. The guided questions asked about stakeholders’ perceptions and experiences of the R&D program under the CPAs and the contribution of the program to community pharmacy practice and the healthcare system (see appendix 1). Each interview was conducted by two investigators, AH/ES or AH/IK. All interviews were audio-recorded and transcribed verbatim. The interviews continued until data saturation was reached, when no new themes or information emerged.

Complete transcripts of the interviews were analysed iteratively together with the audio recordings. This study employed an inductive approach to the meaning of the data, and themes were constructed without pre-determined topics. Several transcripts that were considered to have particular richness of information were selected to create the coding framework, which was constructed collaboratively by all investigators. Data were initially broadly categorized into an initial coding scheme, the codes were clustered into categories, and the categories classified into themes and sub-themes. This technique allowed the investigators to modify the coding framework and add new themes as they emerged from the data.

### Theoretical approaches

There are numerous models, theories or frameworks analysing variable factors contributing to successful implementation of an intervention or research [38–40]. However, the application of such models, theories or frameworks depends on the objective, context, interaction of actors and the complexity of the system in which the research is conducted. The Promoting Action on Research Implementation in Health Services (PARIHS) framework was developed as a tool to explain the success or failure of implementation programs [41–43], and was considered appropriate for analysis of the multi-dimensional elements of knowledge translation in community pharmacy practice.

The PARIHS framework suggests that successful implementation of Evidence Based Practice (EBP) is a function of the relationship among Evidence (E), Context (C) and Facilitation (F). Evidence in the PARIHS framework can be derived from a variety of sources particularly research, clinical experience, patient experience and local data/information. Context refers to the environment or setting in which the research is to be implemented which is influenced by economic, social, political, historical, fiscal and psychological factors. Furthermore, the PARIHS framework defines culture, leadership, monitoring and evaluation as central to determining the context for change. Facilitation in the PARIHS framework relates to processes which enable the implementation of evidence into practice. Within the facilitation element, facilitators, who can be individuals or teams, from internal or external sources, play key roles in affecting the context in which the research is implemented and with their skills, knowledge and roles, help other individuals, teams or organisations to apply the evidence into practice.

The three core elements (E, C, F) are dynamic, equal and simultaneously interrelated. Each element encompasses a range of potentially applicable conditions or sub-elements that determines the status of the three core elements on a weak (low level) to strong (high level) continuum. The framework uses a three dimensional matrix to show that the three core elements can influence the implementation in either a positive (high: H) or negative (low: L) way [41]. Assuming that high quality evidence is available, (notwithstanding that low evidence may be useful in conditions where other elements are favorable), the matrix demonstrates that successful implementation of an innovation is most likely to occur when the context is supportive of change and there is strong facilitation for change. In contrast, less successful implementation is most likely when the context is not receptive to change and there is inadequate facilitation. In a condition when one of the two elements, for instance, context, is low, it may be overcome by the appropriate facilitation or vice versa. This implies that improvement, for example, in infrastructure may be required to change the context or staff development and training is perhaps needed to ensure appropriate facilitation of the innovation.

## Results

A total of twenty-seven key stakeholders participated in the interviews between December 2014 and August 2015 (Table 2).

**Table 2:** Characteristics of participants.

Characteristics	n = 27
Male, n (%)	20 (74)
Background of profession, n (%)	
Pharmacy practitioners and managers	8 (30)
Other healthcare professionals	1 (4)
Academics and researchers	3 (11)
Policy makers and administrators	13 (47)
Consumer representatives	1 (4)
Insurance providers	1 (4
State, n (%)	
ACT	3 (11)
NSW	12 (45)
QUE	4 (15)
SA	2 (7)
VIC	6 (22)
Urban area, n (%)	24 (88)
Method of interview, n (%)	
Face to face	14 (52)
Over the phone	7 (26)
Skype^®^ video call	6 (22)
Average duration of interview (min)	71 min (range 43–93 min)

Three broad themes were identified from the interviews: the value and role of R&D, the operation of the R&D program, and the uptake and challenge for effective implementation.

### The value and role of R&D

Participants expressed the view that investment in the R&D program under the CPAs was essential for developing and strengthening community pharmacy practice. R&D has been recognized as an important element for driving innovation and the long term incremental quality improvement of services provided in community pharmacy.

*“All services that we have today have the seed planted maybe 20 years ago with some R&D*. *You just don’t wake up and roll out that service so I think there has to be the seed somewhere*, *it has to be developed and it has the fruit so I would say R&D is integral part of the long-term strategy”* (P01_MP). **Value of R&D in driving innovation**.

More importantly, the R&D program generated evidence that was critical to demonstration of the efficacy of changes in community pharmacy, and thus to act as a driver of change.

*“Unbelievably important*. *I mean it’s what we use as the reason why we make a decision to implement a service…We need to show evidence of*, *particularly when it’s not widespread and we don’t have it across every pharmacy in Australia*, *we need to continue that research and development”* (P06_MP). **R&D as driver for change**.

### The operation of R&D program

A range of views was expressed about the way the program operated, and particularly how the funding model changed across successive agreements. Several participants in this study expressed concerns about the small quantum of money allocated for funding R&D especially under the 5^th^ CPA. Moreover, the trend towards funding commissioned projects was criticized as it limited opportunities for innovation.

*“We haven’t seen the development of new services or better way of doing things as a result because they have tailored*, *also we have seen net reduction in amount of investment in R&D as a total proportion of the funding and that very much has been tailored to answering questions that the stakeholder on the agreement wants to know rather than what might be something that is more useful broadly and might be needed”* (P02_FP). **Shifted funding towards Commissioned project and small proportion for funding R&D**

Some participants expressed their disappointment with the discontinuation of funding under the 6^th^ CPA. One participant questioned the source for pharmacy to innovate and change practice in the future when there is no funding available for R&D.

*“I think it’s disappointing*. *It doesn’t appear to be in the agreement*. *I think over the successive agreements R&D has been one of the key drivers of improvement and the evolution of new services and it’s very disappointing it’s not there so where does funding for research into pharmacy then come from”* (P08_FP). **Discontinuation of R&D program under the 6**^**th**^ **CPA**

However, according one participant, rather than continuing to fund new R&D, the purpose of the 6^th^ CPA is to generate high level evidence to facilitate implementation of CPS which have already been developed through R&D in previous agreements.

*“This agreement has ceased funding for the research and development program*. *However*, *instead of doing the R&D program*, *that’s where the $50 million trial programs are going to come into play…under the Sixth Agreement what you might actually do is continue on with that work*, *but rather than funding it as an R&D project*, *you might actually fund it as an implementation pilot and trial”* (P25_MNP). **Funding trial of R&D projects under the 6**^**th**^ **CPA**

### The uptake and challenge for effective implementation

The majority of participants perceived that CPS had been widely adopted by community pharmacy in daily practice but that the quality and consistency of provision have been variable. Few participants claimed to know whether the provision of CPS led to better health outcomes for patients.

*“If you measure the signup rate then they were really well adopted because every pharmacy is registered to record clinical interventions*, *to receive payment for Dose Administration Aids and so on*. *If you think about whether they are regularly and genuinely delivering services like Medscheck and making a difference to the people who are they delivered to*, *I think that is a different question and I am not sure we can answer the question with data we have available”* (P02_FP). **Uptake of R&D into practice**

Some participants stated that provision of CPS has contributed to more income for their pharmacy, however, the income gained from providing CPS is much smaller than income from dispensing.

*“The more you drive the professional income*, *the bigger your wages budget you'll get*…*but it is still a very*, *very small part compared to dispensing*, *and in a lot of our business we have a strong*, *very strong retail offer but compared to our dispensing it is still very small part”* (P22_FP). **Profitability for delivering CPS**

Furthermore, a number of factors that act as barriers to implementation were identified. One participant mentioned the lack of patient demand, pharmacists’ ability to deliver, and funding as barriers that need to be addressed, and pointed out that all were important in the successful implementation of CPS.

*“There’s an implementation barrier… You need the patient demand*, *you need a pharmacist’s ability to deliver on that demand*, *and then you need a funding for that position*, *or the owner that will take the lead and do that*. *So you could have the two of those and without the third it’s not going to happen*. *So yeah*, *we could have the funding*, *we could have the pharmacist ready to deliver*, *but if you don’t have the patient demand yeah*, *I guess we’re not going to see that”* (P06_MP). **Barriers in Effective Implementation**

Reflecting on the case of DMAS and PAMS, where the demonstration of high quality evidence provided a strong argument in favour of ongoing funding, the difficulties associated with service practicalities were raised by some participants as a key counter argument to continuation of funding.

*“With those two particular programs*, *there was a strong case that they were valuable services*. *However*, *I think in their conception they were over engineered*. *There was a reliance with the DMAS on absolute cooperation with GPs*, *with HbA1C readings*. *If you couldn’t engage with the doctors*, *then you couldn’t access the doctors*. *So it was in some ways the design was a fail*. *The principle behind it was very good*. *The same with the Asthma thing that involved some spirometry and some training”* (P20_MP). **Practicability of R&D project**

The majority of participants argued that R&D projects must be able to demonstrate cost savings along with the improved clinical outcome to justify further implementation. One researcher involved in HMR posited that HMR was adopted for implementation as it has evidence of both clinical benefits and cost savings for government.

*“We did that original research (and) we are able to demonstrate there is benefit that outweighs the cost and that's the reason they got out*. *And sure that was good thing for pharmacists to do but ultimately for government they looked at the benefit outweighing cost*, *they're looking for savings*. *So to me the fact the way I actually did that work and we're able to demonstrate the value was just*, *was probably the factor that allows us to get up”* (P09_MP). **Cost Saving value of R&D Project**

Furthermore, some participants suggested that patient acceptance was a determining factor for whether a R&D project was funded for widespread implementation. Patients were often unaware of the role of pharmacists in delivering the new CPS and the benefit gained from the provision of the CPS, and thus chose not to access the services.

*“There’s been examples of failed programs where the program has looked great on paper*, *but there’s actually been very low consumer subscription because they don’t necessarily perceive that as being the role of a pharmacy or as a pharmacist”* (P25_MNP). **Patient’s acceptance**

In the end, the majority of participants indicated that political commitment had the greatest influence in deciding whether a CPS achieved ongoing funding or not. Despite strong research evidence of efficacy, it was essentially political agreement among the involved stakeholders within the decision-making process that determined whether or not further implementation occurred.

*“I think at the end of the day it came to what the Minister was more interested in*. *Although that’s not what they’re saying now but the reality is that how it was…I don’t believe that it’ll be totally evidence based now*, *I reckon it’s going to be money driven as well*. *But the other thing is that it’s negotiated by two parties*, *the Guild and the Government*, *and unless there’s perceived to be a groundswell interest the warmth of the Guild to negotiate for anything is going to sort of drive what happens*. *If the Guild doesn’t perceive that pharmacy is interested in doing it*, *then they’re not going to argue for it and in my experience the Government has not yet ever said well this is what we need to do”* (P27_FP). **Political supports in decision making process**

## Discussion

This study confirms that the R&D program under the consecutive CPAs has played an important role in driving some changes through innovation and development of new services in Australian community pharmacy. The CPS generated from the R&D program have created an impetus for role expansion of community pharmacy, and in general, stakeholders had positive views about the value of R&D programs and their benefit to community pharmacy practice. Without R&D projects, it was perceived to be very difficult for community pharmacy to generate evidence that demonstrates value for money of the CPS which is critical to securing funding within the CPAs. Whilst the provision of CPS provides an additional revenue stream to that provided by dispensing PBS medicines, to date, the revenue has been insufficient to foster a major overhaul of the pharmacy business model based on dispensing to a health services focus.

Using the PARIHS framework described earlier in this paper, the influence of the multiple factors at play (evidence, context, facilitation) in determining which CPS are implemented and in what manner is explored. According to this framework, evidence plays a key role in determining the effective implementation and this is a central element in our findings. With respect to evidence, participants described it as a critical factor for successful adoption of research into practice. The fact that several CPS funded under the CPAs were generated from the R&D program has supported this notion. However, at the same time there is a range of factors relating to context and facilitation, which shape the ways evidence is—and is not—translated into general practice including practicability, incentives for delivery of CPS, patient acceptance, cost saving and value to healthcare and political support for implementation as identified in this study. The PARiHS framework provides an excellent tool for explaining the interplay of these three factors.

Evidence collected under carefully controlled conditions, such as in the R&D programs, is therefore essential for supporting practice change. However, the views of participants were strongly supportive of the notion that evidence should not be restricted to that described in research or controlled trials alone. Practical evidence as a mean to explore practicability of a research which involves exploration and consideration of both clinical and patient experiences of a particular service, is also vital in determining the likelihood of successful implementation. With particular reference to DMAS and PAMS, it is clear that the research evidence that was generated indicated that the programs resulted in clinical, economic and humanistic outcomes being met when the context was supportive. Within small scale controlled trials, the impact of many practical variables was able to be minimized, and the support and facilitation provided by the research team was instrumental in maintaining the necessary incentive to continue. However, wider scale rollout resulted in more variability within the context, and a dilution of the effect of facilitation as pharmacists were required to maintain their own motivation to continue. With respect to the DMAS a lack of interest from patients in combination with time and capacity constraints of the pharmacists, rather than any deficiency in the program itself, was responsible for the lower than expected uptake of the service [19]. Likewise, despite high level satisfaction among pharmacists and patients with PAMS research, the decision to cease funding for further PAMS research also played a key role in undermining the potential for knowledge translation [26]. Therefore, participants in this study viewed that knowledge translation resulting from the R&D program was critically dependent on context and facilitation, rather than on the evidence alone, as outlined in the PARiHS model.

Importantly however, the participants also identified a wider range of aspects of context and facilitation, consistent with prior research, contributing to the translation of evidence into practice. A number of studies into the broad area of practice change in community pharmacy have highlighted enthusiasm both from pharmacy stakeholders and policy makers as fundamental to the adoption of changes in practice. [44–47]. This is consistent with the PARIHS framework, since high commitment and receptivity to change are key contextual facilitators, and these were very apparent in the initial phases of both DMAS and PAMS where many pharmacists eagerly signed on to participate in both stages of the national pilot. However, our findings also highlighted a range of barriers to practice change in pharmacy, from both internal and external contexts, as reported in many other studies. These include lack of pharmacists’ capacity due to increased workload [48], poor patient demand [49], limited practicability of the research [50], and lack of incentive for providing the services [51]. In addition, transformation of DMAS into Medscheck suggested that while an evidence based CPS may be refashioned, it is fundamental to maintain its core components—the aspects with proven effectiveness—in any new services. The core components must be implemented with high fidelity and remain untouched while allowing for adjustment for non-core components to adapt with the context or local needs. Furthermore, the small investment in R&D programs and remuneration for CPS under the consecutive CPAs has corresponded to slow practice change in most community pharmacies. As a result, participants perceived that to date, provision of CPS has not been able to significantly contribute to the viability of community pharmacies which remain highly reliant on income from dispensing. This highlights the need for *a priori* funding of research based on principles of implementation science to inform a strategic approach to knowledge translation of evidence based CPS.

Perhaps an important aspect contributing to facilitation and context is the political commitment from peak pharmacy organisations and the government supporting practice change. For instance, the Pharmacy Guild of Australia and the Australian government influenced practice change through negotiation of the successive CPAs within which billions of dollars have been invested including for the R&D program and payment for delivering CPS. However, the community pharmacy sector is a complex and dynamic system with influential elements interacting at the micro, meso and macro level [52]. In this type of system, indirect or less obvious facilitators must also be taken into account. For example, despite not being involved directly in the negotiation for the CPAs, other organisations have been able to push their change agenda in parallel. One example, is the role of the Pharmaceutical Society of Australia in facilitating community pharmacy change through education and targeted practice programs which may or may not receive recognition from the policy perspective. Within this complex system the activities of different pharmacy groups, have been influential, albeit often indirectly or weakly [53]. Gauging their relative influence on practice change is difficult, however, what is clear is that in order to be effective the various pharmacy organisations need to work in unison with other health professions and the consumer to drive practice change [53,54].

A further impact of government policy as facilitator and constraint can be seen in several programs that have been funded in recent CPAs, and which have little or no research evidence to support them. This has been particularly apparent in the decision to fund CPS such as Medscheck, Diabetes Medscheck (in itself a much abbreviated version of DMAS) and Clinical Interventions, despite these services not being supported by any solid evidence for better health outcomes in the context of the Australian healthcare setting [32]. Although like HMR, these services were capped near the conclusion of the 5^th^ CPA, their introduction clearly demonstrates that evidence is not the only factor considered by policy makers.

This study clearly identified that in the contemporary context, all three aspects of the PARIHS model must be taken into account when attempting to understand why some programs are successfully adopted and others are not. The use of the PARIHS framework demonstrates that change is a process resulting from the interaction of evidence, context and facilitation, and provides a plausible explanation of the current situation. Despite sound evidence of the potential for efficacy from the DMAS and PAMS research programs, constraints tended to exceed facilitators within a context which was already resistant to change. Political policy based on pressure to create financial savings, poor patient understanding of the potential roles of pharmacists in broader health care, limitations associated with pharmacists themselves, the siloed nature of the health care system, and a willingness of policy makers to downplay the value of evidence combined to overshadow evidence. These multiple factors demonstrated that decision making for adopting research into practice is a “complex, messy and demanding task” [55].

One of the lessons of this study is that policy makers might be more willing to favour funding of CPS when there is evidence of significant potential savings to the healthcare system such as reduced medication costs (i.e. reduced claims of PBS dispensing), reduced number of hospitalizations, or reduced number of doctors’ visits. With rising healthcare expenditure, governments are concerned to curb the growth in expenditure without adversely affecting health outcomes. However, for the research to be successfully translated and services to be delivered by pharmacists, it is also important to take account of the economic/business implications for pharmacies and whether or not pharmacists perceive that they have the capacity to implement the CPS. In other words, pharmacists need to believe that implementing practice change to focus on delivery of CPS is going to be economically beneficial to ensure the sustainability of the practice, the investment for the pharmacy and income of the individual pharmacist(s).

The PARIHS framework has often been used to analyse knowledge translation in a small scale setting such as a company, organization or work division. The utilization of the PARIHS framework in a broader context as proposed in this study is a novel discussion and some important insights were gained when the framework was used to meet the aim of this study. Furthermore, this study used empirical data in the form of opinions from the stakeholders to seek to explain the relationship between the framework and the real world situations. It confirms that utilization of the PARIHS framework might be applicable as a strategy for analyzing actual cases.

### Strength and limitations

This study included a wide range of key stakeholders with particular experiences and views about the R&D program in community pharmacy. While we may not necessarily have captured the full spectrum of behaviors, attitudes and influences in policy making and implementation, the diversity of opinions elicited nevertheless reflected the complexity and multifactorial nature of influences on knowledge translation in community pharmacy, a topic which has not been widely explored in the current pharmacy literature. There have been some policy changes since the completion of data collection for this study, including the commencement of the Pharmacy Trials Program under the 6th CPA and the ongoing review on pharmacy remuneration and regulation, which to some extent have raised questions about the contribution of R&D programs to community pharmacy services. Nevertheless, these two policies were excluded from consideration as they took effect after 1 July 2015. These policies undoubtedly will have an impact in the future implementation of R&D funded services in community pharmacy.

## Conclusion

This paper summarizes the perceptions and experiences of key stakeholders regarding the operation of an R&D program funded under the CPAs in Australian pharmacy, and its significance in fostering practice change. While R&D programs have been viewed as essential for supporting changes in community pharmacy practice through development and funding of CPS, the overall impact has been small, as contemporary practice continues to be predominantly a dispensing model. The utilization of the PARIHS framework in this study also served to shed light on the complex relationship between evidence, context and facilitation and CPS funding policy decisions and subsequent knowledge translation into community pharmacy practice.

## Supporting information

S1 File Interview guide. (DOCX)

## References

[1] Brooks JM, Doucette WR, Wan S, Klepser DG. Retail Pharmacy Market Structure and Performance. Inquiry. 2008;45(1):75–88. doi: 10.5034/inquiryjrnl_45.01.75 18524293 10.5034/inquiryjrnl_45.01.75

[2] Anscombe J, Thomas M, Plimley J. The Future of Community Pharmacy in England. London: AT Kearney, 2012.

[3] Singleton JA, Nissen LM. Future-proofing the pharmacy profession in a hypercompetitive market. Research in social & administrative pharmacy. 2014;10(2):459. 23820045 10.1016/j.sapharm.2013.05.010

[4] Scahill S, Harrison J, Carswell P, Shaw J. Health care policy and community pharmacy: implications for the New Zealand primary health care sector. NZ Med J. 2010;6. 20657630

[5] Chapman C, Braun L. The professional pharmacist and the pharmacy business. Australian Prescriber. 2011;34(2):34–5.

[6] Richardson E, Pollock AM. Community pharmacy: moving from dispensing to diagnosis and treatment. BMJ: British Medical Journal. 2010;340(7755):1066–8. 10.1136/bmj.c2298 20460333

[7] Roberts AS, Benrimoj SIC, Chen TF, Williams KA, Hopp TR, Aslani P. Understanding practice change in community pharmacy: a qualitative study in Australia. Research in social & administrative pharmacy: RSAP. 2005;1(4):546–64. 17138495 10.1016/j.sapharm.2005.09.003

[8] Hopp TR, Klinke BO, Sørensen EW, Herborg H, Roberts AS. Implementation of cognitive pharmaceutical services in Danish community pharmacies—perceptions of strategists and practitioners. International Journal of Pharmacy Practice. 2006;14(1):37–49.

[9] Feletto E, Wilson LK, Roberts AS, Benrimoj SI. Flexibility in community pharmacy: a qualitative study of business models and cognitive services. Pharmacy World & Science. 2010;32(2):130–8. 20016934 10.1007/s11096-009-9355-3

[10] Australian National Audit Office. Administration of the Fifth Community Pharmacy Agreement. Canberra: ANAO, 2015.

[11] Calder R, Bentley K, Moore K. Evaluation of the Third Community Pharmacy Agreement Research and Development Grants Program. Canberra: NOVA Public Policy, 2006 April 2006. Report No.

[12] Pharmacy Guild of Australia. The Fourth Community Pharmacy Agreement Research & Development Program Overview and Findings. Canberra: PGA, 2010.

[13] Mitchell B, Armour C, Lee M, Song YJ, Stewart K, Peterson G, et al Diabetes Medication Assistance Service: The pharmacist's role in supporting patient self-management of type 2 diabetes (T2DM) in Australia. Patient Education and Counseling. 2011;83(3):288–94. doi: 10.1016/j.pec.2011.04.027 21616627 10.1016/j.pec.2011.04.027

[14] Saini B, Krass I, Armour C. Development, Implementation, and Evaluation of a Community Pharmacy—Based Asthma Care Model. Annals of Pharmacotherapy. 2004;38(11):1954–60. doi: 10.1345/aph.1E045 15479780 10.1345/aph.1E045

[15] Krass I. Pharmacy Diabetes Care Program Final Report. Sydney: Faculty of Pharmacy The University of Sydney, 2005.

[16] Krass I, Armour CL, Mitchell B, Brillant M, Dienaar R, Hughes J, et al The Pharmacy Diabetes Care Program: assessment of a community pharmacy diabetes service model in Australia. Diabetic medicine: a journal of the British Diabetic Association. 2007;24(6):677–83. 17523968 10.1111/j.1464-5491.2007.02143.x

[17] Department of Health and Ageing. Diabetes Pilot Program—Diabetes Medication Assistance Service (DMAS) Stage 1 Implementation Trial. Sydney: Department of Health and Ageing the Australian Government, 2010.

[18] Krass I, Mitchell B, Song YJC, Stewart K, Peterson G, Hughes J, et al Diabetes Medication Assistance Service Stage 1: impact and sustainability of glycaemic and lipids control in patients with Type 2 diabetes. Diabetic Medicine. 2011;28(8):987–93. doi: 10.1111/j.1464-5491.2011.03296.x 21418096 10.1111/j.1464-5491.2011.03296.x

[19] Hales J, Alderdice A, Staniford T, Manser J. Evaluation of The Diabetes Pilot Program Final Report. South Australia: Health Outcomes International, 2010.

[20] The Pharmaceutical Society of Australia. Guidelines for pharmacists providing medicines use review (MedsCheck) and diabetes medication management (Diabetes MedsCheck) services. In: Australia TPSo, editor. Canberra: The Pharmaceutical Society of Australia; 2012.

[21] Krass I. Feature—Diabetes: Optimising Pharmacy Support in Type 2 Diabetes: Lessons from the DMAS. AJP: The Australian Journal of Pharmacy. 2010;91(1086):39–40.

[22] PricewaterhouseCoopers. Combined Review of Fifth Community Pharmacy Agreement Medication Management Programmes. Canberra: PWC, 2015.

[23] Armour C, Bosnic-Anticevich S, Brillant M, Burton D, Emmerton L, Krass I, et al Pharmacy Asthma Care Program (PACP) improves outcomes for patients in the community. Thorax. 2007;62(6):496–592. doi: 10.1136/thx.2006.064709 17251316 10.1136/thx.2006.064709 PMC2117224

[24] Saini B, LeMay K, Emmerton L, Krass I, Smith L, Bosnic-Anticevich S, et al Asthma disease management—Australian pharmacists’ interventions improve patients’ asthma knowledge and this is sustained. Patient Education and Counseling. 2011;83(3):295–302. doi: 10.1016/j.pec.2011.05.001 21621947 10.1016/j.pec.2011.05.001

[25] Emmerton LM, Smith L, LeMay KS, Krass I, Saini B, Bosnic-Anticevich SZ, et al Experiences of community pharmacists involved in the delivery of a specialist asthma service in Australia. BMC health services research. 2012;12(1):164-. 22709371 10.1186/1472-6963-12-164 PMC3439711

[26] Wallace A, Wilkinson R, Benton M, Spinks J, Keevy N, Jones L. Evaluation Of The Asthma Pilot Program (Stage 1) Final Evaluation Report. Canberra: Urbis, 2010.

[27] Roughead EE, Semple SJ, Gilbert AL. Quality use of medicines in aged-care facilities in Australia. Drugs & aging. 2003;20(9):643–53. 12831289 10.2165/00002512-200320090-00002

[28] Gowan J. Home Medicine Reviews and the Aged. Journal of Complementary Medicine: CM, The. 2006;5(2):30–95.

[29] Hilmer S, Chen T, Castelino R, Nishtala P, Bajorek B. Drug Burden Index and Potentially Inappropriate Medications in Community-Dwelling Older People The Impact of Home Medicines Review. 2010. 10.2165/11531560-000000000-00000 20104939

[30] Stafford A, Tenni P, Peterson G, Doran C, Kelly W. The Economic Value of Home Medicines Reviews (IIG-021-VALMER) Tasmania: University of Tasmania, 2009.

[31] Tenni P. HMRs-Status Quo or Quo Vadis? Australian Pharmacist. 2011;30(7):596.

[32] Gilbert AL. Time to Rethink the Funding Model for Home Medicines Review. Journal of Pharmacy Practice and Research. 2014;44(1):9–11.

[33] Gilbert A, Rigby D. HMRs: Is the Damage out of Control? Journal of Pharmacy Practice and Research. 2013;43(1):4–7.

[34] Maher JH, Lowe JB, Hughes R, Anderson C. Understanding community pharmacy intervention practice: lessons from intervention researchers. Research in social & administrative pharmacy: RSAP. 2014;10(4):633–46. 24231257 10.1016/j.sapharm.2013.09.002

[35] Almarsdóttir AB, Kaae S, Traulsen JM. Opportunities and challenges in social pharmacy and pharmacy practice research. Research in social & administrative pharmacy: RSAP. 2014;10(1):252–5. 23652025 10.1016/j.sapharm.2013.04.002

[36] Dobrow MJ, Goel V, Upshur REG. Evidence-based health policy: context and utilisation. Social Science & Medicine. 2004;58(1):207–17. 10.1016/s0277-9536(03)00166-7 14572932

[37] Glasgow RE, Emmons KM. How can we increase translation of research into practice? Types of evidence needed. Annual review of public health. 2007;28(1):413–33. 10.1146/annurev.publhealth.28.021406.144145 17150029

[38] Ellen M. Knowledge Translation Framework for Ageing and Health: A Framework for Policy Development. World Health Organization, 2012.

[39] Moullin JC, Sabater-Hernández D, Fernandez-Llimos F, Benrimoj SI. A systematic review of implementation frameworks of innovations in healthcare and resulting generic implementation framework. Health research policy and systems / BioMed Central. 2015;13(1):16-. 10.1186/s12961-015-0005-z PMC4364490 25885055

[40] Patwardhan PD, Amin ME, Chewning BA. Intervention research to enhance community pharmacists' cognitive services: a systematic review. Research in social & administrative pharmacy: RSAP. 2014;10(3):475–93. 24071523 10.1016/j.sapharm.2013.07.005

[41] Kitson A, Harvey G, McCormack B. Enabling the implementation of evidence based practice: a conceptual framework. Quality in health care: QHC. 1998;7(3):149–58. 10185141 10.1136/qshc.7.3.149 PMC2483604

[42] Ullrich PM, Sahay A, Stetler CB. Use of Implementation Theory: A Focus on PARIHS: Use of Implementation Theory. Worldviews on Evidence-Based Nursing. 2014;11(1):26–34. doi: 10.1111/wvn.12016 24103045 10.1111/wvn.12016

[43] Harvey G, Kitson A. PARIHS revisited: from heuristic to integrated framework for the successful implementation of knowledge into practice. Implementation science. 2016;11:33 doi: 10.1186/s13012-016-0398-2 27013464 10.1186/s13012-016-0398-2 PMC4807546

[44] Noyce PR. Providing Patient Care Through Community Pharmacies in the UK: Policy, Practice, and Research. The Annals of Pharmacotherapy. 2007;41(5):861–8. doi: 10.1345/aph.1K015 17456540 10.1345/aph.1K015

[45] Mossialos E, Naci H, Courtin E. Expanding the role of community pharmacists: policymaking in the absence of policy-relevant evidence? Health policy (Amsterdam, Netherlands). 2013;111(2):135–48. 10.1016/j.healthpol.2013.04.003 23706523

[46] Strand MA, Miller DR. Pharmacy and public health: a pathway forward. Journal of the American Pharmacists Association. 2014;54(2):193 doi: 10.1331/JAPhA.2014.13145 24584835 10.1331/JAPhA.2014.13145

[47] Mossialos E, Courtin E, Naci H, Benrimoj S, Bouvy M, Farris K, et al From "retailers" to health care providers: Transforming the role of community pharmacists in chronic disease management. Health policy (Amsterdam, Netherlands). 2015;119(5):628. 10.1016/j.healthpol.2015.02.007 25747809

[48] Lounsbery JL, Green CG, Bennett MS, Pedersen CA. Evaluation of pharmacists' barriers to the implementation of medication therapy management services. Journal of the American Pharmacists Association: JAPhA. 2009;49(1). 10.1331/JAPhA.2009.017158 19196597

[49] Carter SR. Consumers' willingness to use Home Medicines Review: Faculty of Pharmacy; 2012.

[50] Peterson GM, Jackson SL, Fitzmaurice KD, Gee PR. Attitudes of Australian pharmacists towards practice-based research. Journal of clinical pharmacy and therapeutics. 2009;34(4):397–405. doi: 10.1111/j.1365-2710.2008.01020.x 19583672 10.1111/j.1365-2710.2008.01020.x

[51] McMillan SS, Wheeler AJ, Sav A, King MA, Whitty JA, Kendall E, et al Community pharmacy in Australia: a health hub destination of the future. Research in social & administrative pharmacy: RSAP. 2013;9(6):863. 23218552 10.1016/j.sapharm.2012.11.003

[52] Hermansyah A, Sainsbury E, Krass I. Investigating influences on current community pharmacy practice at micro, meso, and macro levels. Research in Social and Administrative Pharmacy. 2016. 10.1016/j.sapharm.2016.06.007 27530306

[53] Zellmer WA. The Role of Pharmacy Organizations in Transforming the Profession: The Case of Pharmaceutical Care. Pharmacy in History. 2001;43(2/3):75–85. 11837273

[54] Ferguson O. The role of international pharmacy organisations. Pharmaceutical Journal. 2000;264(7077):27–9.

[55] Rycroft-Malone J. The PARIHS framework—a framework for guiding the implementation of evidence-based practice. Journal of nursing care quality. 2004;19(4):297–304. 15535533 10.1097/00001786-200410000-00002

