# Supplementary material for: The operation of a Research and Development (R&D) program and its significance for practice change in community pharmacy
Source: PLoS One. 2017 Sep 18;12(9):e0184954. doi: 10.1371/journal.pone.0184954 (PMC5602630; doi:10.1371/journal.pone.0184954)
Supplement: S1 File — (DOCX) [file pone.0184954.s001.docx]

**Interview guide**

1. How do you view the current situation in Australian Community Pharmacy?

*Delivery of CPS in community pharmacy*

1. What are your opinions on each of the following CPS funded under the CPA:
2. Home Medicine Reviews;
3. Dose Administration Aids;
4. Medscheck and Diabetes Medscheck;
5. Clinical Intervention?
6. How well the CPS have been adopted?
7. What do you think of the way they are structured?
8. What do you think of the contribution of CPS to practice of community pharmacists and viability of community pharmacy? What about the impact to healthcare system?

*The administration of R&D program under the consecutive CPAs*

1. What do you think of the key argument to actually fund specific CPS in the CPA? How important to have evidences for these services?
2. What is your opinion about funding for R&D under the CPA? Do you think it has been effective to support evidence for professional services?
3. What do you think of the relevance between R&D and practice?
4. What do you think of programs which have been trialed such as DMAS and Asthma services? What were the reasons they were not funded for further implementation?
5. Specific for DMAS, how do you compare DMAS and Diabetes Medscheck today? What are the reasons DMAS becoming truncated into Medscheck Diabetes?
